# A super-resolution method-based pipeline for fundus fluorescein angiography imaging

**DOI:** 10.1186/s12938-018-0556-7

**Published:** 2018-09-19

**Authors:** Zhe Jiang, Zekuan Yu, Shouxin Feng, Zhiyu Huang, Yahui Peng, Jianxin Guo, Qiushi Ren, Yanye Lu

**Affiliations:** 1 Department of Biomedical Engineering, College of Engineering, Peking University, Beijing, 100871 China; 2 Hospital Affiliated to Xuzhou Medical University, Xuzhou, 221006 China; 3 School of Electronic and Information Engineering, Beijing Jiaotong University, Beijing, 100044 China; 4 Pattern Recognition Lab, Friedrich-Alexander-University Erlangen-Nuremberg, 91058 Erlangen, Germany

**Keywords:** Fundus fluorescein angiography imaging, Super-resolution, Machine learning, Random forest, Convolutional network

## Abstract

**Background:**

Fundus fluorescein angiography (FFA) imaging is a standard diagnostic tool for many retinal diseases such as age-related macular degeneration and diabetic retinopathy. High-resolution FFA images facilitate the detection of small lesions such as microaneurysms, and other landmark changes, in the early stages; this can help an ophthalmologist improve a patient’s cure rate. However, only low-resolution images are available in most clinical cases. Super-resolution (SR), which is a method to improve the resolution of an image, has been successfully employed for natural and remote sensing images. To the best of our knowledge, no one has applied SR techniques to FFA imaging so far.

**Methods:**

In this work, we propose a SR method-based pipeline for FFA imaging. The aim of this pipeline is to enhance the image quality of FFA by using SR techniques. Several SR frameworks including neighborhood embedding, sparsity-based, locally-linear regression and deep learning-based approaches are investigated. Based on a clinical FFA dataset collected from Second Affiliated Hospital to Xuzhou Medical University, each SR method is implemented and evaluated for the pipeline to improve the resolution of FFA images.

**Results and conclusion:**

As shown in our results, most SR algorithms have a positive impact on the enhancement of FFA images. Super-resolution forests (SRF), a random forest-based SR method has displayed remarkable high effectiveness and outperformed other methods. Hence, SRF should be one potential way to benefit ophthalmologists by obtaining high-resolution FFA images in a clinical setting.

## Background

Fundus fluorescein angiography (FFA) imaging is a valuable diagnostic tool for many ocular and systemic diseases including malarial retinopathy, glaucoma, malignant hypertension and multiple sclerosis [1–4]. Ocular lesions can be detected in the early phases by examining FFA images, which improves the chances of early diagnosis of some diseases, such as, age-related macular degeneration (AMD) [5] and diabetic retinopathy (DR) [6]. Therefore, FFA images can provide ophthalmologists with additional data that can ultimately improve a patient’s cure rate. For example, diabetic retinopathy, which is the leading cause of blindness in adults world-wide [7], can be treated effectively via laser surgery if diagnosed at an early stage. Although routine checkups with ophthalmoscopes have already promoted early diagnosis of ocular diseases, the overall diagnostic rate is still limited by the shortage of ophthalmologists and optometrists [8] in most countries. Hence, high-resolution (HR) FFA images become a vital tool in ophthalmopathy screenings because the HR images can provide doctors with more indicators, such as micro-aneurysms, hemorrhages, and small veins, to assist their diagnosis decision. However, only low-resolution (LR) FFA images are available in most clinical cases. Thus, super-resolution (SR) techniques that aim at enhancing the spatial resolution of images by using image-processing techniques have great potential to improve ophthalmic disease diagnosis rates.

SR techniques were pioneered by Tsai and Huang in 1984 [9] and are mainly used to improve nature and remote sensing images. Generally, there are two major categories of SR algorithms, multi-frame SR and single image SR (SISR). The early SR methods are mainly multi-frame SR [10–13]. The core idea of such methods is an algorithm that combines the information of a sequence of LR images to construct a correlated HR image. On the other hand, the SISR methods focus on learning the relationship between HR and LR images from a training set and recover a correlated HR image from a single LR image. Benefitting from the development of machine learning techniques, various SISR algorithms [14–25] have been proposed in recent years and have become the main research direction of SR algorithms.

In recent years, SR techniques have successfully been extended to medical imaging applications; this provides an important preprocessing step that can improve the image quality of imaging technologies such as ultrasound [26, 27], CT [28], PET [29] and MRI [30–35]. There is also relevant research on using SR techniques on conventional fundus images. Thapa et al. evaluated several SR algorithms and demonstrated the promising impact of partial SR methods [36]. However, the study of Thapa et al. is limited by the low amount of experimental data.

Considering the limitations of current methods, FFA images provide more valuable clinical information than conventional fundus images. Thus, applying super-resolution methods to FFA images can help ophthalmologists achieve better diagnosis results. To the best of our knowledge, no one has applied SR techniques to FFA imaging so far; thus, we propose a SR method-based pipeline for FFA imaging. The aim of this pipeline is to enhance the image quality of FFA by using super-resolution techniques. In this study, four types of SISR algorithms will be analyzed, i.e., neighborhood embedding (NE) [14, 15] approaches, sparsity-based approaches [16, 17], locally-linear regression approaches [18–21] and deep learning (DL)-based approaches [22, 23]. We investigate the effectiveness of each method using our clinical FFA datasets. The results of each algorithm are then quantitatively evaluated to investigate the method’s feasibility and performance.

## Methods

### Super-resolution method-based pipeline

**Fig. 1:**
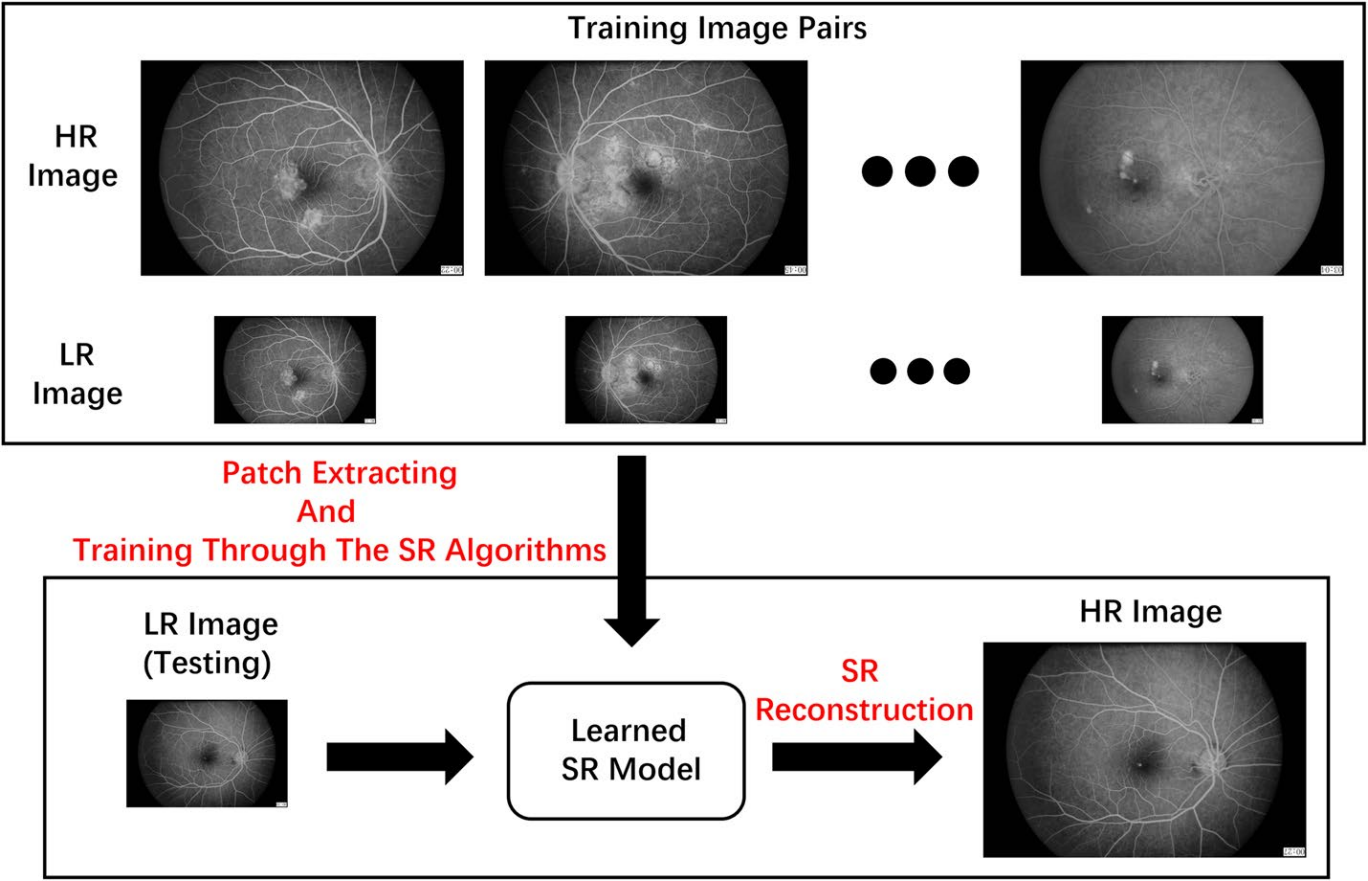
A schematic diagram of the proposed SR method-based pipeline

As an important branch of SR, the SISR method mainly depends on machine learning techniques and shows promise in the field of SR research. Hence, we propose an SR-based pipeline for FFA image enhancement based on SISR methods. A typical schematic diagram of the pipeline is illustrated in Fig. 1. First, an FFA training set that includes HR and LR FFA image pairs is constructed and translated into the form of patch pairs (details will follow shortly); then, a mapping model between the HR images and the LR images can be learned from the training set by using SISR methods; finally, new HR FFA images can be reconstructed from their correlated LR FFA images using the model found in the learning stage of the process. Considering most SISR methods are applicable to the proposed pipeline, we divide the methods into four categories and investigate the most promising techniques to evaluate the feasibility of the pipeline. In this section, we briefly describe the processing process and the parameter setting process of the ten testing SISR algorithms.

Before describing each process, we define the mathematical notation we use in this paper (mathematical definition and details will follow shortly). For the training phase, a set of *patch pairs* are extracted from the original training image pairs. This yields the training set ${\mathcal{P}} = \left\{ {{\mathcal{X}}_{h} ,{\mathcal{X}}_{l} } \right\} = \{ \left( {x_{l}^{n} ,x_{h}^{n} } \right)|n = 1,2, \ldots ,N\}$, where ${\mathcal{X}}_{h} = \left\{ {x_{h}^{1} ,x_{h}^{2} , \ldots ,x_{h}^{N} } \right\}$ and ${\mathcal{X}}_{l} = \left\{ {x_{l}^{1} ,x_{l}^{2} , \ldots ,x_{l}^{N} } \right\}$ are a set of HR and LR image patches (with *N* samples), respectively. Meanwhile, we denote $X_{h} = \left[ {x_{h}^{1} ,x_{h}^{2} , \ldots ,x_{h}^{N} } \right] \in R^{{S_{h} \times N}}$ and $X_{l} = \left[ {x_{l}^{1} ,x_{l}^{2} , \ldots ,x_{l}^{N} } \right] \in R^{{S_{l} \times N}}$ as the matrix representation of the two sets, where $S_{h}$ and $S_{l}$ are the dimensions of the HR and LR patches in vector form, respectively. For the testing phase, we denote the testing LR image patch by $y_{l} \in R^{{S_{l} \times 1}}$ and the reconstruction HR image patch by $y_{h} \in R^{{S_{h} \times 1}} .$

The parameter restrictions of the patch extraction are shown in Table 1, which will be used in the following algorithm instructions and experiments. In this work, the parameter settings of each algorithm are based on values recommended by a set of relevant papers and partial parameters are modified empirically to improve our FFA images. From Table 1, we can see that DL-based approaches (SRCNN [22] and VDSR [23]) and non-DL approaches [14–21] adopt a variety of patch extraction schemes. On one hand, the non-DL approaches densely extract small patch pairs from the training image pairs with an overlap between adjacent patches; the size of LR and HR patches need to be restricted by a fixed scale-based upscaling factor. On the other hand, the two testing DL-based approaches first interpolate the LR images to standardize the size of the original training image pairs with the corresponding HR images. Then large patch pairs, with or without slight overlap, are extracted from the training image pairs. Finally, according to the specific network design (e.g., whether the zero-padding is used for convolutional layers), the border of the HR patches is cropped to constitute the final training patch pairs.

**Table 1:** Parameter setting for the patch-extraction of ten test SISR algorithms on the pipeline (M represents the upscaling factor of the SR task)

	Patch size (LR patch)	Patch size (HR patch)	Sampling stride (LR patch)	Sampling stride (HR patch)
Non-DL methods [14–21]	3 × 3 pixels	3M × 3M pixels	2 pixels	2M pixels
SRCNN [22]	33 × 33 pixels	21 × 21 pixels	14 pixels	14 pixels
VDSR [23]	41 × 41 pixels	3 × 3 pixels	41 pixels	41 pixels

#### Neighborhood embedding approaches

As a representative of learning-based SISR methods, the neighborhood embedding (NE) method was proposed by Chang et al. [14] in 2004. The NE method assumes that LR and HR patches naturally lie on local manifolds with a locally similar geometry in feature space. Once sufficient training samples are obtained, patches in the HR feature space can be reconstructed via a linear combination of local neighbors using the same weights learned in the corresponding LR feature space. Thus, for each input LR patch $y_{l}$, the NE method first determines the K-nearest patches in the training pool ${\mathcal{X}}_{l}$ by calculating the Euclidean distance to get a subset of nearest neighbors ${\mathcal{N}}_{knn} = \left\{ {x_{lk}^{1} ,x_{lk}^{2} , \ldots ,x_{lk}^{K} } \right\}$, where $x_{lk}^{i}$ is a selected patch from ${\mathcal{X}}_{l}$ and

1 $$ N_{knn} = {\mathop {\arg \hbox{min} }\limits_{{x_{l}^{n} \in X_{l} }}}^{K} ||y_{l} - x_{l}^{n} ||_{2}^{2} . $$

The input LR patch $y_{l }$ can now be approximated by using a weighted combination of the learned subset of nearest neighbors ${\mathcal{N}}_{knn}$:

2 $$ y_{l} \approx \sum\limits_{{x_{lk}^{i} \in N_{knn} }} {w_{i} x_{lk}^{i} = N_{knn} {\mathbf{w}}} , $$

where $N_{knn} = \left[ {x_{lk}^{1} ,x_{lk}^{2} , \ldots ,x_{lk}^{K} } \right] \in R^{{S_{l} \times K}}$ and **w** is the vector of $\left\{ {w_{i} } \right\}_{i = 1}^{K} .$

Considering that the local manifold assumption is used by all kinds of NE methods, the means of solving the weight coefficient $\{ w_{i} \}_{i = 1}^{K}$ are essential. In this work, we implement neighborhood embedding with least squares (NE + LS) [14] and neighborhood embedding with non-negative least squares (NE + NNLS) [15]; these methods employ least squares algorithms and non-negative least squares constraints to calculate the set of weights **w**. Therefore, the weight-calculation of the NE + LS and NE + NNLS methods can be expressed as

3 $$ {\mathbf{w}} = \mathop {\arg \hbox{min} }\limits_{{\mathbf{w}}} ||y_{h} - N_{knn} {\mathbf{w}} | |_{2}^{2} \quad {\text{s}} . {\text{t}} .\quad 1^{\text{T}} {\mathbf{w}} = 1 $$

and

4 $$ {\mathbf{w}} = \mathop {\arg \hbox{min} }\limits_{{\mathbf{w}}} ||y_{h} - N_{knn} {\mathbf{w}} | |_{2}^{2} \quad {\text{s}} . {\text{t}} .\quad {\mathbf{w}} \ge 0, $$

respectively.

Once the weight coefficients are determined, they can be used to reconstruct the HR patch $y_{h}$ based on the nearest neighbor subset ${\mathcal{H}}\left( {{\mathcal{N}}_{knn} } \right) = \left\{ {x_{hk}^{1} ,x_{hk}^{2} , \ldots ,x_{hk}^{K} } \right\}$, which is the counterpart of ${\mathcal{N}}_{knn}$ for the HR patch space ${\mathcal{X}}_{h} .$

5 $$ y_{h} = \sum\limits_{{x_{hk}^{i} \in H(N_{knn} )}} {w_{i} x_{hk}^{i} } . $$

In the proposed pipeline, the size of the nearest neighbor set K, of both NE + LS and NE + NNLS, was set to 24.

#### Sparsity-based approaches

Sparsity-based approaches [16, 17] are different from NE-based approaches. The former needs to learn subsets of patches (${\mathcal{N}}_{knn}$ and ${\mathcal{H}}\left( {{\mathcal{N}}_{knn} } \right)$) for every input LR patch from the entire LR and HR training patch spaces (${\mathcal{X}}_{l}$ and ${\mathcal{X}}_{h}$) are used to reconstruct HR patches; the latter integrates sparse coding [37] into the SR problem and aims at simplifying the patch spaces by learning the compact dictionaries. As a pioneer, Yang et al. [16] proposed training a coupled dictionary pair, which can characterize the LR and HR patch spaces with the same sparse representation. Given the training set ${\mathcal{P}} = \left\{ {{\mathcal{X}}_{h} ,{\mathcal{X}}_{l} } \right\},$ the coupled dictionary-based joint training problem is defined as

6 $$ \{ D_{h} ,D_{l} ,Z\} = \mathop {\text{arg min}}\limits_{{{\{ D_{h} ,D_{l} ,Z\}}}} \frac{1}{{S_{h} }}||X_{h} - D_{h} Z||_{2}^{2} + \frac{1}{{S_{l} }}||X_{l} - D_{l} Z||_{2}^{2} { + }\lambda \left( {\frac{1}{{S_{h} }} + \frac{1}{{S_{l} }}} \right)||Z||_{1} , $$

where $D_{h} \in R^{{S_{h} \times B}}$ and $D_{l} \in R^{{S_{l} \times B}}$ are the LR and HR dictionaries, respectively. The scalar B is the number of dictionary atoms, and $Z \in R^{B \times N}$ is the encoding coefficient that couples both HR and LR dictionaries. Once the $D_{h}$ and $D_{l}$ have been trained, the reconstruction of input LR patch $y_{l}$ can be formulated as Eqs. 7 and 8.

7 $$ \alpha = \mathop {\arg \hbox{min} }\limits_{\alpha } ||y_{l} - D_{l} \alpha ||_{2}^{2} + \lambda ||\alpha ||_{1} $$

8 $$ y_{h} = D_{h} \alpha . $$

The sparse representation α ($\upalpha \in R^{B \times 1}$) of $y_{l}$ is firstly calculated by minimizing Eq. 7, where the regularization parameter λ balances the importance of the sparsity constraint. Then the reconstructed HR patch $y_{h}$ is obtained directly via matrix multiplication of the sparse representation α and the HR dictionary *D*_*h*_.

Zeyde et al. [17] have improved the training scheme based on [10]. By reformulating the joint training of coupled dictionaries into two consecutive optimization problems (Eqs. 9, 10), the dictionaries $D_{l}$ and $D_{h}$ can be retrieved by applying the K-SVD [38] algorithm and pseudo-inverse techniques, respectively. In the reconstruction phase, the so-called orthogonal matching pursuit (OMP) [39] is further applied to facilitate the solving procedure of the sparse representation *α* in Eq. 7. Finally, the HR patch $y_{h}$ can be reconstructed based on Eq. 8.

9 $$ \{D_{l}, Z\} = \mathop {\text{arg min}}\limits_{{{\{D_{l} , Z\}}}} ||X_{l} - D_{l} Z||_{\text{F}}^{2} \quad {\text{s}}.{\text{t}}.\quad ||z^{n} ||_{0} \le L\quad for\quad n = 1,2, \ldots ,N $$

10 $$ D_{h} = \mathop {\text{arg min}}\limits_{{D_{h} }} ||X_{h} - D_{h} Z||_{\text{F}}^{2} $$

In this work, we used SB-Yang and SB-Zeyde to signify the two sparsity-based approaches, respectively. Both approaches set the number of dictionary atoms and regularization parameter λ to 2048 and 0.1, respectively. The parameter *L* for the solution process of K-SVD is set to 24 atoms for each representation vector.

#### Locally-linear regression approaches

Combining the ideas of both the NE-based approaches and the sparsity-based approaches, Timofte et al. proposed a locally-linear regression approach called anchored neighborhood regression (ANR) [18]. In the training phase, this method employs the SB-Zeyde technique to train the coupled dictionaries $D_{h}$ and $D_{l}$. Two significant modifications were introduced to the reconstruction procedure. First, the global dictionaries are subdivided into various sub-dictionaries. Then, the LR dictionary $D_{l}$ with B dictionary atoms can be represented as $D_{l} = \left[ {d_{l}^{1} ,d_{l}^{2} , \ldots ,d_{l}^{B} } \right]$, where $d_{l}^{j} \left( {{\text{j}} \in \left[ {1,{\text{B}}} \right]} \right)$ is one dictionary atom of $D_{l}$. For each $d_{l}^{j}$, a corresponding sub-dictionary $N_{la}^{j} = \left[ {d_{la}^{1\left( j \right)} ,d_{la}^{2\left( j \right)} , \ldots ,d_{la}^{K\left( j \right)} } \right] \in R^{{S_{l} \times K}}$ is constructed with the k-nearest neighbors from the dictionary atoms of $D_{l}$. Then, by locating the counterpart of $N_{la}^{i}$ in the HR dictionary $D_{h}$, the HR sub-dictionaries $N_{ha}^{j} = \left[ {d_{ha}^{1\left( j \right)} ,d_{ha}^{2\left( j \right)} , \ldots ,d_{ha}^{K\left( j \right)} } \right] \in R^{{S_{h} \times K}}$ can be also obtained. Moreover, the ANR algorithm uses the *L*_*2*_-norm constraint of the coefficient matrix instead of the *L*_*1*_-norm constraint for the sparse representation; this is done to simplify the optimization problem to a ridge regression [40], which can be solved in closed-form. Therefore, for the input LR patch $y_{l}$ with the nearest dictionary atom $d_{l}^{j}$, the optimization problem of Eq. 7 can be reformulated (as Eq. 11) by combining the sub-dictionaries and *L*_*2*_-norm regularization

11 $$ \beta = \mathop {\arg \hbox{min} }\limits_{\beta } ||y_{l} - N_{la}^{j} \beta ||_{2}^{2} + \lambda ||\beta ||_{2} , $$

which has a closed-form solution

12 $$ \beta = ((N_{la}^{j} )^{T} N_{la}^{j} + \lambda I)^{ - 1} (N_{la}^{j} )^{T} y_{l} . $$

Then, the reconstructed HR patch can be denoted as

13 $$ y_{h} = N_{ha}^{j} \beta = [N_{ha}^{j} ((N_{la}^{j} )^{T} N_{la}^{j} + \lambda I)^{ - 1} (N_{la}^{j} )^{T} ]y_{l} = P_{j} y_{l} , $$

where $P_{j}$ is the so-called projection matrix for the *j*-th dictionary atom, which can be calculated offline. For each input LR patch $y_{l}$, the reconstruction procedure of ANR can be simplified to find the nearest-neighbor atom $d_{l}^{j}$ for $y_{l}$ in the LR dictionary $D_{l}$ and using the corresponding projection matrix $P_{j}$ to finish the SR reconstruction via the matrix multiplication of $P_{j}$ and $y_{l}$.

Depending on the simplified architecture of ANR, Timofte further proposed an adjusted anchored neighborhood regression (A+) [19]. A+ inherits various tricks of ANR, such as sub-dictionary and *L*_*2*_-norm regularization; but for A+, the training samples are no longer discarded after training the coupled dictionaries, whereas ANR and most of the sparsity-based approaches do. Instead, these training samples are directly applied to the reconstruction procedure via the use of sub-dictionaries. For each atom $d_{l}^{j}$ from the LR dictionary $D_{l}$, A+ searches its k-nearest neighbors among the training pool ${\mathcal{X}}_{l}$, instead of the sparse dictionary atoms of $D_{l}$. Therefore, the LR and HR sub-dictionaries of A+ can be denoted as $N_{ls}^{j} = \left[ {x_{ls}^{1\left( j \right)} ,x_{ls}^{2\left( j \right)} , \ldots ,x_{ls}^{K\left( j \right)} } \right] \in R^{{S_{l} \times K}}$ and $N_{hs}^{j} = \left[ {x_{hs}^{1\left( j \right)} ,x_{hs}^{2\left( j \right)} , \ldots ,x_{hs}^{K\left( j \right)} } \right] \in R^{{S_{h} \times K}}$, where $x_{ls}$ and $x_{hs}$ are training samples selected from ${\mathcal{X}}_{l}$ and ${\mathcal{X}}_{h}$ respectively. Based on the solved $N_{ls}^{j}$ and $N_{hs}^{j}$, A+ reconstructs the HR patch using the same method that ANR does.

Unlike ANR and A+, which needs the trained coupled dictionaries to divide the patch spaces and use the dictionary atoms as alternative anchor points for local linear regression, jointly optimized regressors (JOR) algorithms [20] tries to directly learn the separation of the patch spaces and the corresponding regressors by solving a joint optimization problem. For the given training examples ${ \mathcal{P}} = \left\{ {{\mathcal{X}}_{h} ,{\mathcal{X}}_{l} } \right\}$, JOR clusters the training patches into *O* groups and learns *O* regressors ${\mathcal{F}} = \left\{ {f_{1} ,f_{2} , \ldots ,f_{O} } \right\}$, which collectively provide the least reconstruction error for all the training patches (*O* is the fixed number assigned manually). The problem can be expressed as follows:

14 $$ \{ C,\text{F}\} = \mathop {\text{arg min}}\limits_{C,F} \sum\limits_{n = 1}^{N} {\sum\limits_{o = 1}^{O} {c_{o,n} } } ||f_{o} (x_{l}^{n} ) - x_{h}^{n} ||_{2}^{2} , $$

where ${\text{C}} \in R^{O \times N}$ is the cluster indicator of training sample, in which $c_{o,n} = 1$ represents that the training sample $n$ in cluster *O*, otherwise $c_{o,n} = 0$. An iterative algorithm resembling EM algorithm [41] is used to solve this problem. Two procedures (E-step and M-step) are implemented to update the ${\mathcal{F}}$ and ${\text{C}}$ alternately until Eq. 14 convergence. In the E-step, the clusters ${\text{C}}$ are fixed and ${\mathcal{F}} = \left\{ {f_{1} ,f_{2} , \ldots ,f_{O} } \right\}$ is estimated for each cluster. Once again, ridge regression (Eqs. 11–13) is used to learn the regressors. The SR-reconstructed HR patch of regressor $f_{O}$ can be expressed as $\widetilde{{x_{h}^{o,n} }} = f_{o} \left( {x_{l}^{n} } \right) = P_{o} x_{l}^{n} = [X_{h}^{o} \left( {(X_{l}^{o} )^{T} X_{l}^{o} + \lambda I} \right)^{ - 1} (X_{l}^{o} )^{T} ]x_{l}^{n} .$ Here, $X_{l}^{o}$ and $X_{h}^{o}$ are matrices stacked by all the LR patches and the corresponding HR patches from the *O*-th cluster column-wise. In the M step, the regressors ${\mathcal{F}}$ are fixed and the clusters ${\text{C}}$ should be updated. For each training sample pair $\{ x_{l}^{n} ,x_{h}^{n} \}$, the SR reconstruction error of all regressors $\left\{ {f_{o} } \right\}_{o = 1}^{O}$ are calculated according to $e_{o,n} = \;\parallel \widetilde{{x_{h}^{o,n} }} - x_{h}^{n} \parallel^{2}$; the sample pair is then reassigned to the *o*-th cluster with the minimum reconstruction error $e_{o,n}$ to get the new clusters. Once Eq. 14 is solved, the training of JOR is finished. For the testing step, the input LR patch only needs to find its k-nearest neighbors from the training samples and use these neighbors to evaluate the most suitable regressor $f_{o}$ for SR reconstruction.

Inspired by the basic idea of JOR, Schulter et al. [21] proposed a random forest-based approach called super-resolution forests (SRF); this method is used to directly learn a mapping from the LR patch space to HR patch space. Random forests [42] split the training patch space automatically without defining the number of clusters manually. All the trees in SRF are trained independently and the set ${\mathcal{P}} = \left\{ {{\mathcal{X}}_{h} ,{\mathcal{X}}_{l} } \right\}$ includes *N* training samples $\left\{ {x_{l}^{n} ,x_{h}^{n} } \right\}_{n = 1}^{N}$. Moreover, SRF adapts a novel regularized quality measure ${\text{E}}\left( {X_{H} ,X_{L} } \right)$ for the evaluation of splitting functions

15 $$ E(X_{H} , { }X_{L} ) = \frac{1}{|X|}\sum\limits_{n = 1}^{|X|} ( ||x_{h}^{n} - m(x_{l}^{n} )||_{2}^{2} + \kappa ||x_{l}^{n} - \overline{{x_{l} }} ||_{2}^{2} ), $$

where ${\text{m}}\left( {x_{l}^{n} } \right)$ is the HR prediction for the LR sample $x_{l}^{n}$, $\overline{{x_{l} }}$ is the mean value of the $x_{l}^{n}$ in the leaf node and κ is the hyper-parameter. Thus, ${\text{E}}\left( {X_{H} ,X_{L} } \right)$ is a suitable way to efficiently learn the tree structure needed for regression-based SR problem, because it not only promotes the HR prediction precision but also keeps the similarity of the samples from the same leaf node. Once the structure of the tree is fixed, for any leaf node *le* with a linear regression model $m_{le} \left( {x_{l}^{n} } \right) = w^{le} x_{l}^{n}$, SRF can use the training samples $(X_{l}^{le} \;{\text{and}}\;X_{h}^{le} )$, routed to the current leaf node, to calculate the mapping $w^{le}$ via local linear regression. Again, we can get a closed-form solution of $w^{le} = X_{h}^{le} \left( {(X_{l}^{le} )^{T} X_{l}^{le} + \lambda I} \right)^{ - 1} (X_{l}^{le} )^{T} .$ The reconstruction procedure of $y_{h}$ can be implemented by averaging the predictions over all T trees:

16 $$ y_{h} = \frac{1}{T}\sum\limits_{t = 1}^{T} {m_{le(t)} (y_{l} )} , $$

where $l_{\left( t \right)}$ is the leaf node belonging to tree *t* that $y_{l}$ is routed to.

In our pipeline, the ANR and A+ use the trained coupled dictionaries $D_{h}$ and $D_{l}$ to form the SB-Zeyde as the starting point of the algorithm. On the other hand, JOR and SRF directly split the patch spaces without coupled dictionaries. For the ANR and A+ , the weight factors λ of the sparsity constraints were all set to 0.1 and the nearest neighbor size K was set to 40 and 2048 for ANR and A+ , respectively. For JOR, the weight factor λ was fixed to 0.1 and the three main parameters (the number of regressors, the number of iterations of the E-M optimization and the nearest neighbor size K) were set to 32, 20 and 32, respectively. For the SRF case, the parameter settings were the number of trees ${\text{T}} = 6$, the max tree depth $\upxi_{max} = 15$, $\uplambda = 0.1$ and $\upkappa = 1.$

#### Deep learning-based approaches

In recent years, DL has achieved phenomenal success. Various computer vision tasks such as classification, object recognition, and segmentation have benefited from DL’s many functions. Inspired by successful DL models, especially convolutional neural networks (CNN) that are used for classification (such as VGG-Net [43] and ResNet [44]), several CNN-based methods [22–25] were proposed to handle the SISR problem. In this paper, two representative CNN networks for SR, SRCNN [22] and VDSR [23], are implemented in our experiment.

**Fig. 2:**
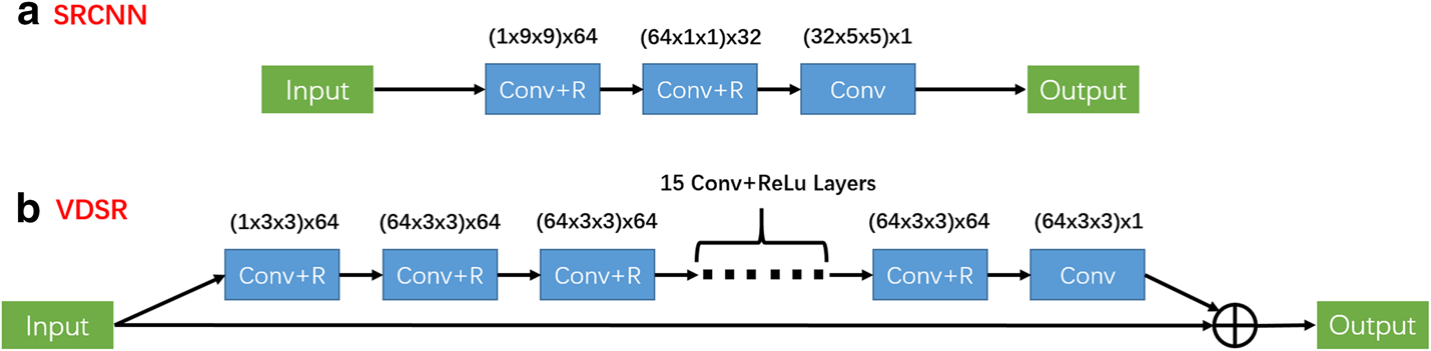
Simplified structures of **a** SRCNN and **b** VDSR

As a pioneer of CNN-based SISR work, SRCNN was proposed by Dong et al. [22] to learn an end-to-end nonlinear mapping from the LR and HR images. A simplified structure of SRCNN is shown in the Fig. 2a, which includes three convolutional layers with filter sizes of 1 × 9 × 9, 64 × 1 × 1 and 32 × 5 × 5. Except for the last layer, rectified linear units (ReLu, ${ \hbox{max} }\left( {0,{\text{x}}} \right)$) [45] are applied following the convolutional layers as the activation function. For the end-to-end system, the network parameters are denoted as $\varTheta = \left\{ {W_{d} ,B_{d} |d = 1,2,3} \right\},$ where $W_{d}$ and $B_{d}$ are the filter weights and biases for the *d*-th convolutional layer. Given the training set ${\mathcal{P}} = \{ \left( {x_{l}^{n} ,x_{h}^{n} } \right)|n = 1,2, \ldots ,N\} ,$ the SRCNN model is estimated by minimizing the mean squared error (MSE) of ground truth HR images $x_{h}^{n}$ and reconstructed HR images ${\text{F}}\left( {x_{l}^{n} ;\varTheta } \right)$. The loss function is characterized by

17 $$ L(\varTheta ) = \frac{1}{N}\sum\limits_{n = 1}^{N} {||F(x_{l}^{n} ;\varTheta ) - x_{h}^{n} ||_{2}^{2} } . $$

The objective function can be minimized by using the stochastic gradient descent (SGD) with the standard backpropagation (BP) [46]. In Dong’s view, the function of the three convolutional layers of SRCNN can be explained in analogy with the pipeline of sparse coding-based SR methods, which includes patch extraction and representation, Non-linear mapping, and reconstruction, respectively. Relying on the highly expressive capability of CNN, SRCNN can explore the nonlinear relationships between the LR and HR images and learn general image representation, which can be applied to various datasets and tasks.

Considering the overall development trend of CNN, that “the deeper the better” in the field of computer vision, Kim et al. proposed a very deep convolution network, termed VDSR. Figure 2b shows the structure of the VDSR, which indicates that VDSR uses 20 weight layers in a cascaded way to form the deep network. Except for the first and last layers, all the weight layers include 64 filters with size 64 × 3 × 3 and with ReLu on filter responses. In this way, VDSR has achieved a significantly larger perspective than SRCNN (41 × 41 vs 13 × 13) to help the network exploit more contextual information to model the SR-mapping tasks. For training, the VDSR adapts the MSE as a loss function and uses SGD with BP to train the network. At the same time, to accelerate the convergence speed of the deep network, Kim also provides several techniques, such as residual learning and adaptive gradient clipping, to ensure the deep network can be trained with a very high learning rate. Residual learning demands that the convolutional layers of VDSR only predict the difference between the LR image and the correlated HR image, i.e., residual images; the LR input image can then be added to the residual image via a skip connection to reconstruct the final HR image. Especially considering that the LR input image and the HR output image are similar in the SR tasks, training a deep convolution network that can predict residual images instead of HR images should be easier to accomplish. Hence, the VDSR has achieved good performance in both training time and reconstruction quality. In fact, nowadays, even if various new CNN models [24, 25], which have more complicated and elaborative designs, are proposed to complete the SR tasks, the VDSR should still be an efficient DL model.

For the training of the SRCNN in the pipeline, the batch size, momentum, and weight decay parameters were set to 128, 0.9 and 0, respectively. The learning rate was 10^−4^ for the first two convolutional layers and 10^−4^ for the third layer. The filter weights were initialized randomly via a Gaussian distribution $(\upmu = 0, \delta^{2} = 0.001)$ and the biases were was initialized with the constant zero. On the other hand, for the training of VDSR, the batch size, momentum and weight decay parameters were set to 16, 0.9 and 0.0001, respectively. The learning rate was initially set to 0.1 and decreased by a factor of 10 every 30 epochs. When the learning rate reached 0.0001, the learning rate stops decreasing and keeps the fixed value in the following epoch. The filter weights are initialized by the method proposed by [47], where the biases were set to 0.

### Experimental setup

A simulation experiment was carried out for quantitative analysis and evaluation of the SISR methods (compared in this work) for the SR method-based pipeline using a clinical FFA dataset. All the experiments were implemented on a workstation (Intel i7-7700 CPU at 3.6 GHz, 32 GB RAM). The non-DL SISR methods were implemented using MATLAB. Meanwhile, DL-based SISR methods (SRCNN and VDSR) are trained using the Caffe package [48] on a GTX 1070 GPU and tested using the MatConvNet package [49].

#### Fundus fluorescein angiography dataset

**Fig. 3:**
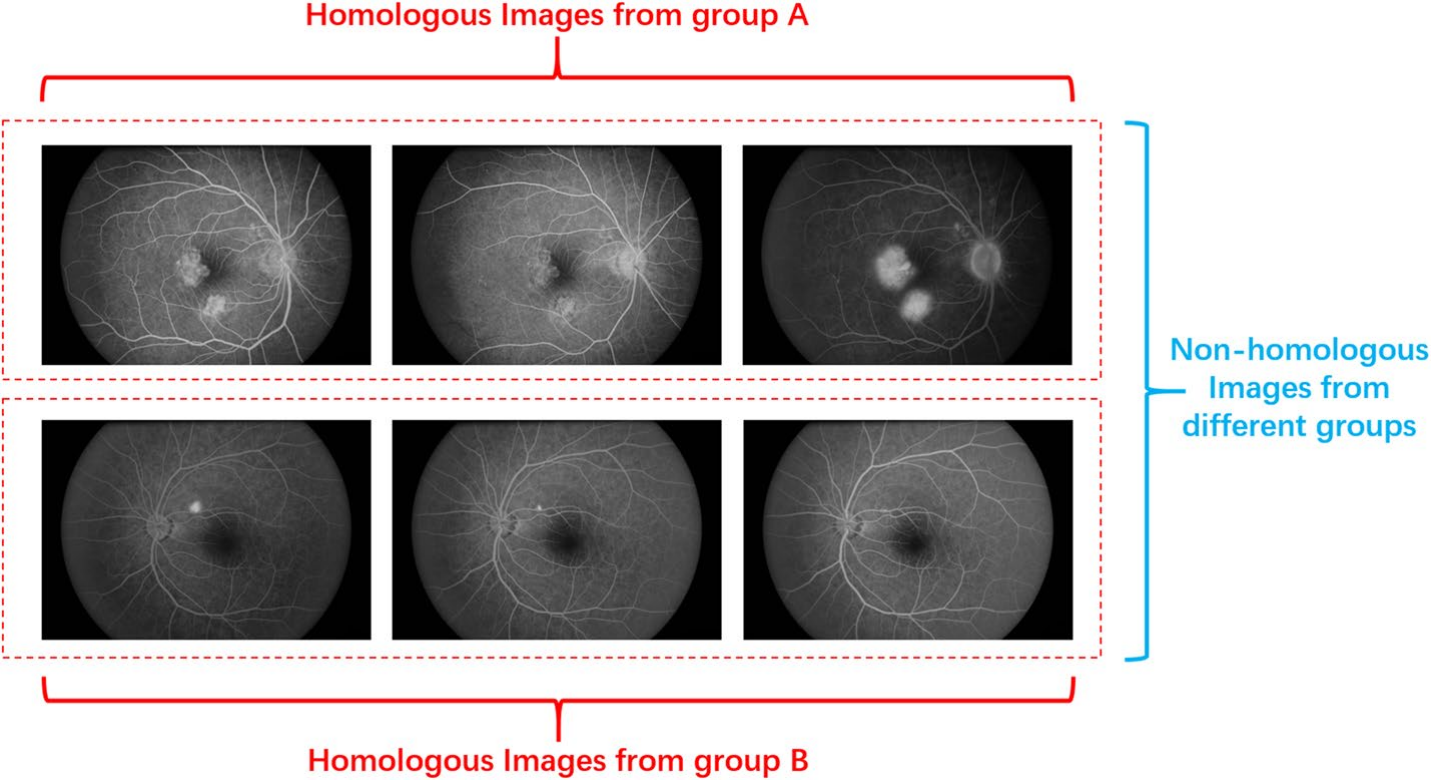
An example of homologous and non-homologous images

To better explore the performance of the pipeline in a clinical setting, we collected 185 FFA images from ten different eyes in Second Affiliated Hospital to Xuzhou Medical University as our dataset. All the FFA images were acquired using Canon (CF-60DSi) equipment. The 185 images have been classified into ten groups. Note that, although the images from the same group were acquired from the same eye, they also have the obvious difference between individuals due to various conditions of translation, rotation, blood flow and lighting distribution. Hence, for convenience, we named the images belonging to the same groups and images belonging to the different groups as the homologous images and non-homologous images, respectively. Figure 3 demonstrates one example to explain the concept of homologous images and non-homologous images.

#### Experimental protocol

The experimental study was performed in accordance with the workflow of the proposed pipeline. We used the original FFA images as the HR images and acquired the corresponding LR images by down-sampling the HR images in the spatial dimension. The down-sampling was done by implementing downsampling factors via a Bicubic downsampler. In this way, the original 185 FFA images were translated into 185 FFA image pairs. The FFA image pairs were divided into a training set TR1 (115 FFA image pairs) and a testing set TE1 (70 FFA image pairs). This is done to ensure that both TR1 and TE1 contain all ten types of homologous images and maintain a unified 23:14 distribution proportion (between TR1 and TE1) for each group of homologous images. Next, we used the HR-LR image pairs from TR1 as the input of the SISR algorithms to train the mapping models. The LR images from TE1 were tested next by using the trained SR models for reconstruction. Finally, the HR images from the TE1 served as the ground truth for quantitative analysis of the reconstruction performance of the SR methods. In this paper, we have performed the experiments using ten representative algorithms under two upscaling factors (2× and 4×) and choose the peak signal-to-noise ratio (PSNR) and structural similarity (SSIM) [50] as the quantitative evaluation indexes.

## Results

Table 2 shows the performance of the ten SISR algorithms using our SR method-based pipeline with two upscaling factors (2× and 4×). The Bicubic interpolation [51] results are also calculated in Table 2 as a baseline to compare the studies.

**Table 2:** The average number of evaluation indexes of SISR algorithms (trained on TR1) for the testing set TE1 (with an upscaling factor: 2×, 4×)

	PSNR		SSIM	
	2×	4×	2×	4×
Bicubic	40.11	34.51	0.994	0.968
NE + LS	42.83	36.16	0.998	0.981
NE + NNLS	42.25	36.10	0.998	0.981
SB-Yang	42.80	36.20	0.998	0.981
SB-Zeyde	43.50	36.60	0.998	0.982
ANR	42.98	36.02	0.998	0.982
A+	43.18	36.97	0.998	0.985
JOR	43.76	37.76	0.997	0.985
SRF	47.06	41.30	0.998	0.986
SRCNN	44.40	37.76	0.997	0.984
VDSR	44.46	38.84	0.998	0.986

**Fig. 4:**
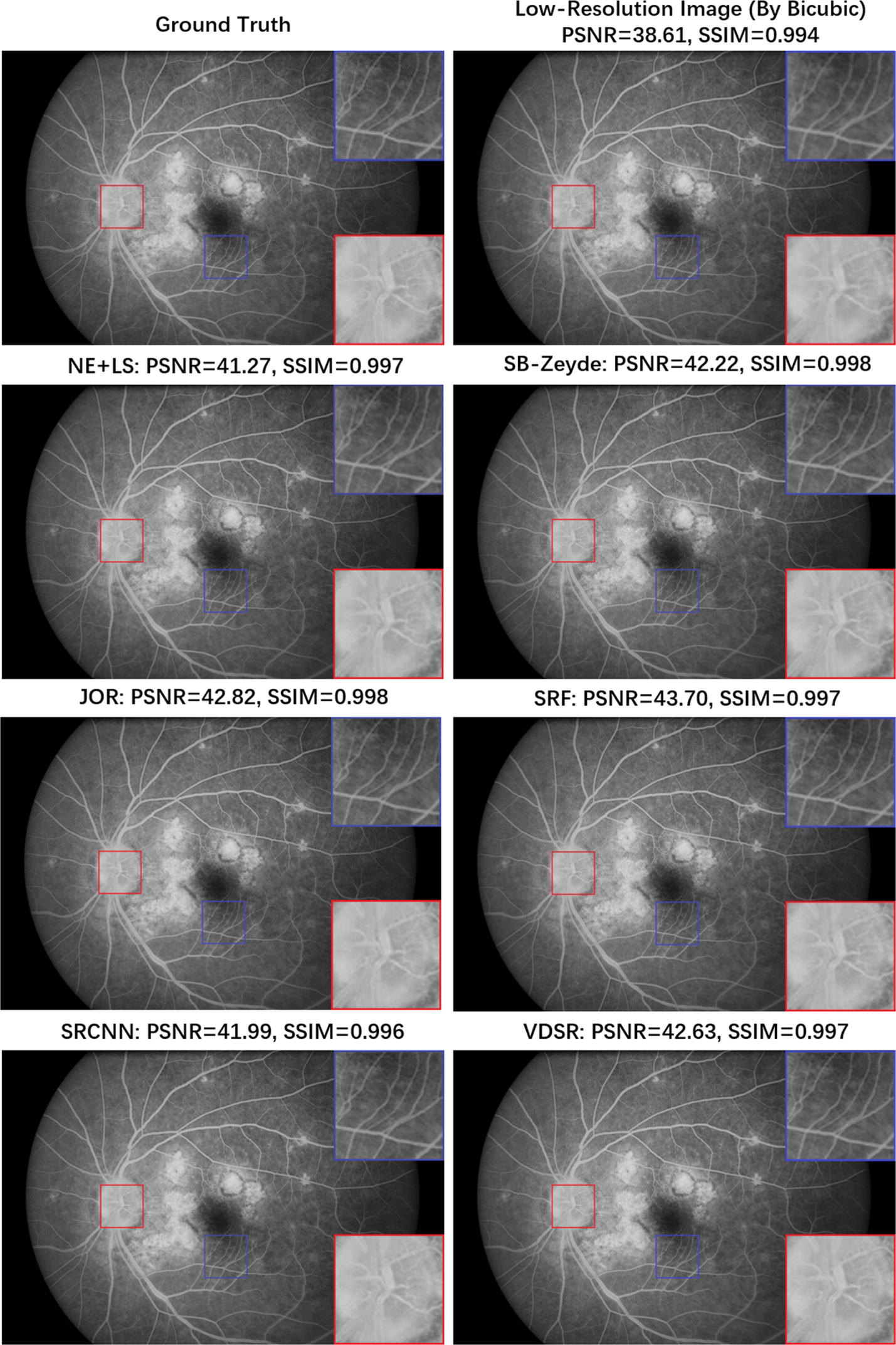
The reconstructed FFA images by different SISR algorithms under the upscaling factor of ×2

**Fig. 5:**
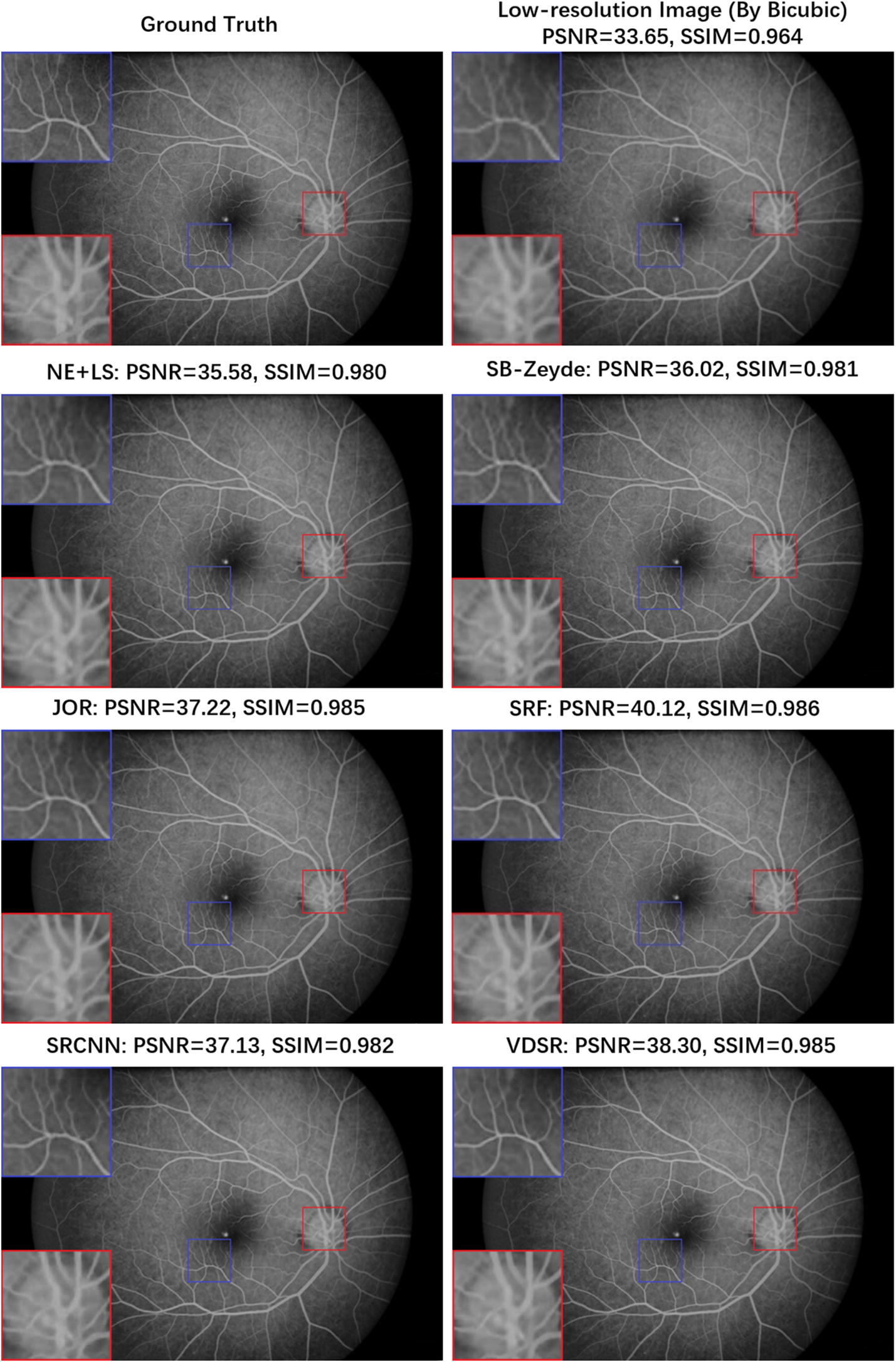
The reconstructed FFA images by different SISR algorithms under the upscaling factor of ×4

As shown in Table 2, all the SR results demonstrate better PSNR and SSIM indices compared to the Bicubic interpolation; this result suggests that the SISR algorithms have successfully extracted suitable feature-mapping models for FFA images from the training set during the image enhancement procedure. For twofold SR tasks, all the SISR algorithms have good evaluation indexes. Figure 4 provides six typical twofold upscaling SR results from the four groups of the SISR algorithms. These SR images have high consistency with the ground truth, and the borderline of the blood vessels among the optic disc and macula region, which are less distinct in the interpolated LR image, can be seen clearly in the SR reconstructed images. On the other hand, while the fourfold SR tasks are more challenging, the evaluation indexes of the reconstructed results have decreased and the performance gap between the Bicubic interpolation and the SISR algorithms becomes more evident compared to the twofold SR tasks. Figure 5 shows the SR results using fourfold magnification. Thus, even under the fourfold upscaling, the locally-linear regression approaches (JOR and SRF) and DL-based approaches (SRCNN and VDSR) still successfully reconstruct most spatial information for the FFA image, particularly in the reconstructed images of SRF and VDSR. The connections of blood vessels at the margins of the optic disc and other important structural details, such as the tiny arteries among the macular region and, can be steadily recovered by the SISR algorithms and this result already provides a similarity to the ground truth. Hence, from both quantitative and visual results, the feasibility of the proposed SR method-based pipeline for FFA imaging has been validated.

We further compare the reconstruction performance of the ten SISR algorithms under two upscaling factors using our FFA datasets. The performance of the SISR algorithms can be roughly ranked by the following order: SRF > VDSR > SRCNN > JOR > A+ > SB-Zeyde > ANR > SB-Yang > NE + LS > NE + NNLS. The ordering shows that the locally-linear regression and DL-based approaches are more effective. The SRF provides the best visual results with the highest PSNR values. Meanwhile, the training time and the average reconstruction speed of the best three SISR algorithms are shown in Table 3. SRF has shown good efficiency with an acceptable reconstruction speed and a significantly shorter training time than the two DL-based methods.

**Table 3:** The training time and averaged reconstruction speed of the best three SISR methods

	SRCNN	VDSR	SRF
Training time (h)	300	41	10
Reconstruction speed (s/sample)	37.2	43.2	57

## Discussion

In this work, we explore the effectiveness of the proposed the proposed SR method-based pipeline for FFA imaging and find the most potential SISR algorithm for clinical practices. From the experimental results, we verified that SRF methods achieved high performance using the FFA dataset. One of the possible reasons for this good performance is that the SRF method can find a suitable number of clusters for the training patch spaces via the building of tree structures. Depending on this efficient division, FFA patches share the similar local fundus features that can be used together to learn more precise locally-linear regression models for the reconstruction of FFA images than other parameter-dependent SISR methods such as sparsity-based approaches, ANR, A+ and JOR. However, to some extent, the superiority of SRF methods over DL-based approaches are unexpected (contrary to the common understanding of the performance of the deep networks). Considering that ten groups of homologous images are divided into both training set and testing set for our experiment, this prior distribution of the homologous images should be a potential influence on the performance of SISR algorithms. In the SRF algorithm, the algorithms are highly-likely to improve the performance via proper regression models determined by the homologous training images of the current test image. Hence, we conduct the second experiment to explore the influence of this prior distribution on the performance of SISR algorithms. In this experiment, we exclude four groups of homologous images from the training set TR1 to obtain the new training set TR2, which contains 52 images from six eyes. Meanwhile, we also reform the testing set TE1 by keeping only four non-homologous groups of images against the TR2 in the testing set. We name this new testing set TE2, which includes 32 images from four eyes. Two best SISR algorithms (SRF and VDSR) in the first experiment are selected for the second experiment. We train these two algorithms based on TR2 and test them on the TE2. For comparison, we also conduct another testing on the TE2 using the trained mapping model (based on TR1) in the first experiment. All the SR procedures in the second experiment use the fourfold upscaling factor. The results are presented in Table 4.

**Table 4:** The average evaluation indexes of SISR algorithms (trained on TR1 and TR2, respectively) for the test set TE2 (with an upscaling factor: 4×)

	PSNR		SSIM	
	TR1	TR2	TR1	TR2
SRF (TE2)	41.86	41.23	0.987	0.986
VDSR (TE2)	39.27	39.00	0.986	0.986

From Table 4, we can see that, although the performances of the two SISR methods decrease without the help of the homologous images in training set, the two SISR algorithms still achieve acceptable results via a trained model of non-homologous images. The SRF has successfully kept the reconstruction quality at a high level. In fact, to reduce the calculation intensity, we have already compromised the partial performance of SRF by simplifying the number of trees of the random forest from 15 (recommended in the original paper) to 6 in our experiments. Even after applying this trade-off, the SRF has “learned” a suitable number of data-dependent regression functions via numerous leaf nodes in the forest for the SR reconstruction of FFA images.

On the other hand, the relatively stable performance of VDSR, shown in Table 4, demonstrates that the training procedure of VDSR is less dependent on the homologous images. One of the possible explanations should be that the deep CNN’s strong capacity for learning and expression can help VDSR extract more general features from the FFA training images to complete the reconstruction. In fact, designing very deep CNN models is a recent trend for SISR algorithms. For example, Mao et al. [52] proposed a 30-layer residual encoder-decoder (RED) networks with symmetric skip connection. Tai et al. later introduced recursive blocks in DRRN (52 layers) [53] and memory blocks in MemNet (80 layers) [54] to construct multi-path deep networks. Li et al. [55] used modified residual blocks to construct EDSR (36 layers) and MDSR (165 layers) for single-scale SR task and multi-scale SR task respectively. All these networks have successfully improved the reconstruction quality of SR images depending on the fine hierarchical features extracted by deep CNN model. However, to make full use of this advantage, large training datasets are usually necessary to avoid the over-fitting problem and improve the final performance of the deep network. This is not the case in this work because the training datasets in our experiment have a limited number of FFA images. On the other hand, high-performance GPUs are another key requirement for the application of deep network to meet the demand of large storage and heavy computation. Considering that many computers in Chinese department of Ophthalmology, especially in primary hospitals, do not have GPUs that can achieve fast calculations of big data sets, the practical applications of the DL-based SISR methods remain limited. Hence, although we realize the potentiality of DL-based SISR algorithms, we believe that SRF should still be the competitive option for the resolution enhancement of FFA images with high generality and usability in a clinical setting at present stage because the algorithm can be efficiently trained on the small size of the training data and the relatively short training and testing time on CPU environment.

Next, we discuss the degradation model of the LR images. In our experiments, we used the degradation model $x_{l} = Gx_{h}$ to simulate the LR FFA images, where $G$ is the downsampling matrix. This degradation model should be treated as a simplified version of the normal degradation model $x_{l} = GB_{u} x_{h}$($B_{u}$ represents the blur matrix). This simplification is made to explore the clinical practice of abandoning images with obvious motion blur and out-of-focus blur that are not used for subsequent diagnosis and analysis. In our experiments, we are concerned with whether our SR algorithm has the capability to recover information from the spatially downsampled FFA images. In fact, the insufficiency of spatial sampling is always a major problem for clinical FFA imaging. On one hand, due to budget constraints, high-performance sensors are not standard equipment for all the clinical environments. In some primary hospitals, the sensor used for FFA imaging have relatively lower spatial resolution and can’t meet the demands of HR imaging. On the other hand, the fluorescence signal of FFA imaging has relative lower intensity than the normal reflective signal of conventional fundus imaging. Additionally, the exposure time can’t be markedly prolonged due to other practical considerations (e.g., eye movement), pixel binning (a kind of downsampling) is often used in clinical settings to increase the signal-to-noise ratio (SNR) of FFA images. Thus, we find that our degradation model of LR images considers common clinical problems. Hence, our simulated LR FFA images should have a certain degree of similarity with LR images typically used in clinics, which also becomes an important guarantee to generalize the experimental results to the clinical practice.

Finally, we believe that the SR-enhanced FFA images are meaningful for ophthalmologists, even if novel imaging modalities such as optical coherence tomography (OCT) have gained great success in recent years. There are three main reasons for our opinion. First, the FFA, as the current gold standard for evaluating the clinical fundus feature of DR and AMD, is still widely used in ophthalmology for diagnosing and classifying related fundus disorders [56–59]. Second, FFA is still the most commonly used method to plan laser treatment (photocoagulation) in clinical settings [60]. Third, for clinical research involving multimodal imaging, OCT, FFA and other modalities are often used cooperatively [61]. In fact, considering the characteristic of OCT images, we also wonder if our proposed SR-based pipeline method can be used to the enhance OCT images, which can be a potential research direction for future work.

## Conclusion

In conclusion, we have preliminary explored the effects of resolution enhancement of the FFA images using an SR-based pipeline method. Ten testing SISR methods, divided into four groups, are used for the proposed pipeline of our clinical FFA datasets. The experimental results are then analyzed and compared. From the results, we find that direct local regression-based approaches and DL-based approaches work well for our (clinical) datasets. Then, as the representative algorithms of these two groups of SISR methods, SRF and VDSR are further discussed on the reformed datasets to discuss the algorithms’ dependency on the training set. Both experimental results have shown that super-resolution method-based pipeline has the potential to enhance FFA images. The SRF has displayed remarkably-high effectiveness and outperformed other testing algorithms. Hence, we believe that the SRF is a feasible SR method that can be implemented on an ophthalmologist’s workstation to create an SR-based pipeline method for FFA images to assist ophthalmologists in enhancing these images in their clinical practices.
